# Osteopontin and Fibronectin Levels Are Decreased in Vitreous of Autoimmune Uveitis and Retinal Expression of Both Proteins Indicates ECM Re-Modeling

**DOI:** 10.1371/journal.pone.0027674

**Published:** 2011-12-14

**Authors:** Cornelia A. Deeg, Christina Eberhardt, Florian Hofmaier, Barbara Amann, Stefanie M. Hauck

**Affiliations:** 1 Institute of Animal Physiology, Department of Veterinary Sciences, Ludwig-Maximilians University, München, Germany; 2 Research Unit for Protein Science, Helmholtz Zentrum München - German Research Center for Environmental Health (GmbH), Neuherberg, Germany; Tulane University, United States of America

## Abstract

Autoimmune uveitis is an intraocular inflammation that arises through autoreactive T-cells attacking the inner eye, eventually leading to blindness. However, the contributing molecular pathomechanisms within the affected tissues remain as yet elusive. The extracellular matrix (ECM) is a highly dynamic structure that varies tremendously and influences the encompassing tissue. In order to assess ECM re-modeling in autoimmune uveitis, we investigated the expression of ECM molecules fibronectin and osteopontin in vitreous and retina samples. This was carried out in the only spontaneous animal model for human autoimmue uveitis, namely equine recurrent uveitis (ERU) that resembles the human disease in clinical as well as in immunopathological aspects. ERU is a naturally occurring autoimmune disease in horses that develops frequently and has already proved its value to study disease-related pathomechanisms. Western blot analysis of fibronectin and osteopontin in healthy and uveitic vitreous revealed significant reduction of both proteins in uveitis. Immunohistochemical expression of fibronectin in healthy retinas was restricted to the inner limiting membrane abutting vimentin positive Müller cell endfeet, while in uveitic sections, a disintegration of the ILM was observed changing the fibronectin expression to a dispersed pattern extending toward the vitreous. Retinal expression of osteopontin in control tissue was found in a characteristic Müller cell pattern illustrated by co-localization with vimentin. In uveitic retinas, the immunoreactivity of osteopontin in gliotic Müller cells was almost absent. The ability of Müller cells to express fibronectin and osteopontin was additionally shown by immunocytochemistry of primary cultured equine Müller cells and the equine Müller cell line eqMC-7. In conclusion, severe ECM re-modeling in autoimmune uveitis reported here, might affect the adhesive function of fibronectin and thus the anchoring of Müller cell endfeet to the ILM. Furthermore, the absence of osteopontin in gliotic Müller cells might represent reduced neuroprotection, an osteopontin attribute that is intensively discussed.

## Introduction

Autoimmune uveitis is a sight-threatening intraocular inflammation driven by eye-invading autoreactive T-cells that cross the blood-retinal barrier [1], [2]. However, the majority of molecular pathomechanisms within the eye contributing to the onset of disease and to the loss of immune-privilege remain as yet unresolved [3]. In order to understand the pathogenesis and the underlying molecular processes of autoimmune uveitis, different experimental animal models are investigated [4]. Among these, equine recurrent uveitis (ERU) is the only spontaneous animal model for autoimmune uveitis, and ERU resembles closely the human disease in many clinical as well as immunopathological aspects [5], [6]. In contrast to the human disease, however, ERU is a frequent pathology in the equine population. Both, the high incidence as well as the close similarity to the human condition, render ERU an ideal tool for studying autoimmune uveitis-related pathomechanisms [7]. The course of the disease is characterized by recurring relapses ultimately leading to tissue destruction and loss of vision [7], [8]. Autoaggressive T-cells that cross the blood-retinal barrier and attack the inner eye are specific for several retinal autoantigens [8], [9]. Among them, cellular retinaldehyde binding protein (CRALBP) was first discovered in ERU [9] and proved subsequently to be a frequently targeted autoantigen in human autoimmune uveitis [10]. The multilayered retina that provides the basis for vision represents the target tissue of autoimmune uveitis [10], [11]. Retinal neuronal cells are heavily influenced by the surrounding extracellular matrix [12]. Since ECM is a highly dynamic structure, the properties can vary tremendously from one physiological state to another [13]. Additionally, ECM molecules of chronically inflamed tissues can be altered by cytokines and proteases that are produced by infiltrating immune cells [14]. ECM does not only fill intercellular space, proteins of the ECM are also involved in a variety of major functions including cellular signalling, regulation of development and differentiation and mediation of cell-matrix adhesion among others [15], [16], [17].

Fibronectin, a glycoprotein of the ECM, mediates cellular-ECM interactions and comprises functions such as cell adhesion, migration, growth and differentiation [18]. It was reported that fibronectin is implicated in adhesion and migration of cultured rat Müller cells [19]. In autoimmune uveitis, Müller cells convert into a gliotic state and change their morphology and function profoundly [20]. Since we found an upregulation of fibronectin in autoimmune uveits in a previous proteomic study comparing the retinal membrane proteome of healthy to uveitic retinas [3], we chose this ECM protein for more detailed investigations.

Osteopontin, a major phosphoprotein of the ECM, has gained interest since reports of its functions associated with inflammation and neuronal disorders are inconsistent. Neuroprotective effects versus proinflammatory properties of osteopontin are discussed. The neuroprotective potential of osteopontin was demonstrated in several studies. Osteopontin treatment of oxygen and glucose deprived cortical neuron cultures protected against cell death and intracerebral ventricular application of osteopontin reduced infarct size in a murine stroke model [21]. Recently, we published a study that confirmed a neuroprotective effect of osteopontin. We showed that osteopontin is derived from retinal Müller cells and has a positive survival effect on cultured primary porcine photoreceptors. Furthermore, we demonstrated a reduced apoptotic rate of photoreceptors in cultured retina explants from a mouse harbouring a mutation resulting in retinal degeneration under the influence of osteopontin [22]. However, proinflammatory features of osteopontin were also shown. Induction of experimental autoimmune uveitis (EAU) in mice with human interphotoreceptor retinoid-binding protein was accompanied with an elevated level of plasma osteopontin, while induction of EAU in osteopontin deficient mice showed milder clinical and histopathological symptoms assuming that osteopontin is proinflammatory [23]. This could be confirmed by downregulation of osteopontin with small interfering RNA in mice with induced EAU [24].

The purpose of this study was to investigate the expression of ECM proteins fibronectin and osteopontin in a spontaneous animal model for autoimmune uveitis to gain further information about these proteins and the role of ECM in a retinal disorder.

## Materials and Methods

### Sample collection and preparation

For this study, a total of 17 ERU diseased specimens and 16 healthy controls were collected and processed. ERU eye tissue specimens derived from horses that were treated in the equine clinic and diagnosed with ERU according to clinical criteria as described [6].

Control samples (retinas, vitreous and posterior eyecups) were obtained from horses euthanized due to incurable and ERU unrelated diseases or where obtained from a local slaughterhouse. Vitreous samples of ERU cases were obtained during therapeutic pars-plana vitrectomy. Uveitic eyes derived from horses that had to be enucleated during a therapeutical procedure. The collection and use of equine eyes derived from horses that were killed due to a research-unrelated cause, was approved for purposes of scientific research by the appropriate board of the veterinary inspection office Munich, Germany (Permit Number: 8.175.10024.1319.3). Horses were treated according to the ethical principals and guidelines for scientific experiments on animals according to the ARVO statement for the use of animals in Ophthalmic and Vision research. No experimental animals were involved in this study.

Vitreous samples were stabilized with EDTA-free protease inhibitor (Roche, Mannheim, Germany), then lyophilized, thereafter solubilised in ultrapure water and dialysed against 50 mM phosphate buffer (pH 7.6). Protein content was determined using the Bradford assay (Sigma-Aldrich, Deisenhofen, Germany).

For immunohistochemistry, posterior eyecups were immersion-fixed with Bouin's solution (Sigma-Aldrich, Deisenhofen, Germany), dehydrated in a series of alcohols and sliced into pre-assigned fragments as described before [25]. Resulting tissue blocks were embedded in paraffin, sectioned (8 µm) and mounted on coated slides (Superforst, Thermo Scientific, Bonn, Germany).

### SDS-PAGE and Western Blotting of vitreous samples

Equal total protein amounts from vitreous samples were separated by SDS-PAGE (8% and 12% gels) and blotted semidry onto polyvinyl difluoride (PVDF) membranes (GE Healthcare, Freiburg, Germany). Unspecific binding was blocked with 1% polyvinylpyrrolidon - tween (PVP-T) for 1 hour at room temperature. Blots were incubated with primary antibodies (anti-osteopontin and anti-fibronectin) at 4°C overnight and then washed 3 times in phosphate buffered saline – tween (PBS-T; 136.9 mM NaCl, 8.1 mM Na_2_HPO_4_×2H_2_O, 1.4 mM KH_2_PO_4_, 2.6 mM KCl, pH 7.2, 0.05% Tween20). Binding was detected by horseradish peroxidise (HRP)-coupled secondary antibody incubation for 1 hour at room temperature. After 6 further washing steps in PBS-T, signals were detected by enhanced chemoluminescence on X-ray films (Fuji; Christiansen, Planegg, Germany). Films were scanned on a transmission scanner and quantification of western blot signals by densitometry was performed using ImageQuantTL software (GE Healthcare). Osteopontin and fibronectin abundances between ERU cases and controls were statistically analyzed using the Mann-Whitney test. The differences in protein expression were considered significant if the significance level was ≤0.05.

### Immunohistochemistry on retina sections

Heat antigen retrieval was performed at 99°C for 15 min in 0.1 M EDTA-NaOH (pH 8.0). To prevent unspecific antibody binding, sections were blocked with 1% BSA in tris buffered saline – tween (TBS-T; 10 mM Tris, 150 mM NaCl, pH 7.2, 0.1% Tween20) containing additionally 5% of a non-immune serum derived from the secondary antibody animal species, for 40 min at RT prior to every primary antibody incubation.

Multiple labelling was performed consecutively, with blocking steps (ProteinBlock; Dako, Hamburg, Germany) between single antibody incubations. For triple labelling, sections were first consecutively incubated with primary antibodies (osteopontin and fibronectin at 4°C, both overnight; Vimentin for 3 hours at RT). Each primary antibody incubation was followed by the respective secondary antibody (30 min at RT). Cell nuclei were counter-stained with 4′,6-diamidino-2-phenylindole (DAPI; Invitrogen, Karlsruhe, Germany; 1∶1000). Finally, the sections were mounted with glass coverslips using Dako fluorescence mounting medium (Dako, Hamburg, Germany). Fluorescent images were recorded with an Axio Imager M1 or Z1 and the Axio Vision 4.6 software (both from Zeiss, Göttingen, Germany) at a total magnification of 40×.

Negative controls for all immunohistochemical experiments included omission of the primary antibody as well as incubation with isotype-matched primary antibody of irrelevant specificity.

### Isolation of equine Müller cells

Primary Müller cells were isolated from healthy equine retinas according to a method described previously [26]. Briefly, vitreous and pigment epithelial attachments were carefully removed from retinas and subsequent disintegration was accomplished mechanically. Retinal dissociation was achieved by treatment with activated papain (Worthington Biochemical Corporation, Troisdorf, Germany) for 30 min at 37°C; reaction was stopped by adding Dulbecco's Modified Eagles Medium (DMEM; Biochrom, Berlin, Germany) containing 10% fetal calf serum (FCS; Biochrom, Berlin, Germany). Desoxyribonuklease (Sigma-Aldrich, Deisenhofen, Germany) was added and resuspended cells were seeded in culture flasks (Cell^+^; Sarstedt, Nümbrecht, Germany) after trituration and centrifugation. The cells were allowed to attach for 24 h at 37°C and 5% CO_2_ in an incubator and non-attached cells were removed. Using the above described method, we previously established the first equine Müller cell line that was named eqMC-7 (equine Müller cell line – 7) [26]. All immunocytochemical experiments were performed on primary cultured equine Müller cells and eqMC-7 cells.

### Immunocytochemistry of equine Müller cells

For immunocytochemistry eqMC-7 cells and primary cultured equine Müller cells were trypsinized and seeded onto glass slides which were placed in culture plates. Cells were cultured in DMEM omitting FCS supplement for 16 h and were allowed to attach to the glass surface within this time. Slides were rinsed in phosphate buffered saline (PBS) and fixed in acetone for 10 min. To prevent unspecific antibody binding, slides were blocked with 1% BSA in TBS-T containing 5% appropriate serum (according to the species the secondary antibody was produced in) for 40 min at room temperature prior to every primary antibody incubation. Fluorescence labelling and image acquisition was performed similarly as for immunohistochemistry of retina sections.

### Antibodies

Goat polyclonal antibody against mouse osteopontin from R&D Systems (Wiesbaden-Nordenstadt, Germany) was used at a dilution of 1∶100 for immunohisto- and immunocytochemical experiments and at a dilution of 1∶500 for western blots. Polyclonal rabbit-anti-fibronectin antibody (Thermo Fisher Scientific, Bonn, Germay) was applied at a dilution of 1∶100 in immunohistochemistry and 1∶400 in immunocytochemistry. It was used at 1∶1000 in western blotting experiments. Monoclonal mouse anti-vimentin antibody (Clone V9) from Sigma-Aldrich (Deisenhofen, Germany) was used at a dilution of 1∶400 in immunohisto- and immunocytochemistry. Alexa Fluor labelled secondary IgG antibodies were obtained from Invitrogen (Karlsruhe, Germany) and all used at a working dilution of 1∶500. We used goat anti-mouse IgG coupled to alexa 488, goat anti-rabbit IgG alexa 647 and donkey anti-goat IgG alexa 546.

## Results

### Osteopontin and fibronectin expression is significantly decreased in uveitic vitreous

**Figure 1:**
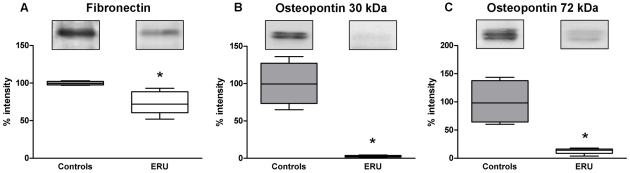
Fibronectin and osteopontin expression in control and uveitic vitreous. Western blot signals for fibronectin (A) and for osteopontin at 30 kDa (B) and at 72 kDa (C) of control (grey boxes, representative band above) and uveitic vitreous samples (white boxes, representative band above) were quantified by densitometry and statistical significance was calculated using the Mann-Whitney test. Data are represented in a box plot and median is marked by a black line in boxes. Expression level of fibronectin (A) was significantly (*p = 0.02) reduced in diseased (n = 4) compared to healthy vitreous (n = 5) with a difference in the median values of 25.8%. Quantification of osteopontin expression at 30 kDa (B) resulted in a significant (*p = 0.02) decrease in uveitic vitreous (n = 4) compared to control samples (n = 5) with the median values of 99.5% (healthy) and 2.8% (diseased). Osteopontin at 72 kDa (C) shows a significantly (*p = 0.02) decreased expression level in uveitic vitreous (n = 4) compared to controls (n = 5). Median value of healthy vitreous was 98% in contrast to 14.3% of uveitic samples.

In order to investigate the occurrence and expression level of fibronectin and osteopontin in vitreous of healthy and ERU diseased eyes, we performed SDS-PAGE and subsequent western blotting experiments. Expression of fibronectin in healthy (Figure 1A, gray box) and diseased vitreous samples (Fig. 1A, white box) was detected at a molecular weight of approximately 260 kDa. Determination and comparison of signal intensities revealed significantly (*p≤0.05) reduced expression levels of fibronectin in vitreous of uveitic eyes (median 74%) compared to control vitreous specimens (median 99.8%). Osteopontin positive reaction was detected in healthy as well as in diseased vitreous samples in form of a double band at the molecular weight of 30 kDa (Fig. 1B) and a band triplet at the molecular weight of 72 kDa (Fig. 1C). Intensities of both signals were determined and compared between healthy (Fig. 1B and C, gray boxes) and uveitic specimens (Fig. 1B and C, white boxes). Signal intensity of the double band at 30 kDa was significantly (*p≤0.05) decreased in vitreous from ERU diseased eyes (median 2.8%) compared to healthy control vitreous (median 99.5%) (Fig. 1B). Quantification of the Osteopontin signal at 72 kDa also revealed a significant reduction in uveitic vitreous (median 14.3%) in comparison to control samples (median 98%) (Fig. 1C).

### Fibronectin immunoreactivity is detectable at the ILM and upregulated in uveitic retinas

**Figure 2:**
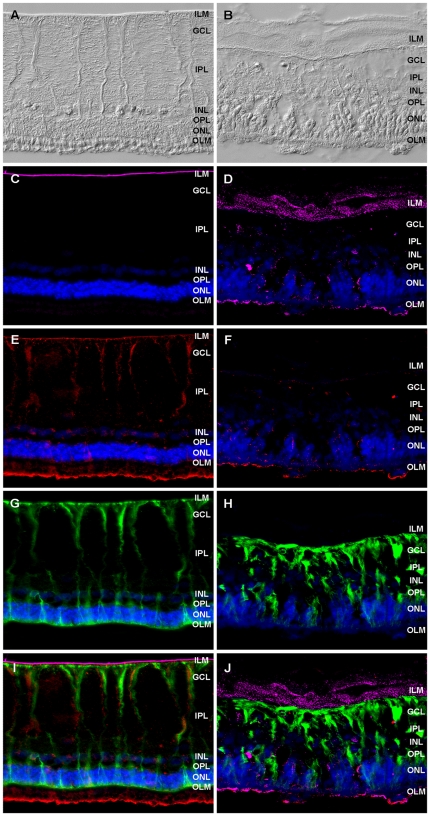
Expression pattern of fibronectin and osteopontin in healthy and uveitic retinal tissue. Retinal expression of fibronectin (magenta), osteopontin (red) and vimentin (green) in a representative healthy (left panels) and ERU diseased retina (right panels). Differential interference contrast (DIC) image of a representative control retina (A) and uveitic retina section (B), display tissue destruction and a loss of photoreceptor outer segments in the ERU case. Fibronectin is expressed along the ILM in healthy retina as a continuous band (C) but shows a spotted pattern along the disintegrated ILM and in the outer uveitic retina (D). Osteopontin expression in healthy retina shows a Müller cell like pattern and a positive staining of photoreceptor outer segments (E). This expression almost disappears in ERU diseased retina (F). Müller cells of control section show a distinct immunoreactivity for vimentin throughout the retina (G) while uveitic retina displays characteristic gliotic Müller cell morphology (H). Overlay image of control retinas reveals a close neighbouring of fibronectin and vimentin at the ILM and an overlap of osteopontin and vimentin especially in Müller cell endfeet (I). The disintegrated fibronectin expression is no longer adjacent to Müller cell endfeet membranes and no co-localization of osteopontin and vimentin is detectable in uveitic retina (J). Nuclei are stained with 4′,6-diamidino-2-phenylindole (DAPI; blue). ILM, inner limiting membrane; GCL, ganglion cell layer; IPL, inner plexiform layer; INL, inner nuclear layer; OPL, outer plexiform layer; ONL, outer nuclear layer; OLM, outer limiting membrane.

In a previous proteomics study comparing the retinal membrane fraction of healthy and uveitic samples we already demonstrated that retinal fibronectin expression is increased in autoimmune uveitis compared to control [3]. To further characterize this extracellular matrix protein we were interested in the expression pattern of fibronectin in the healthy equine retina and whether the retinal distribution would change in autoimmune uveitis. To this end we performed immunohistochemical fluorescent stainings of healthy (Fig. 2, left panels) and uveitic (Fig. 2, right panels) retina sections. Differential interference contrast (DIC) image of control equine retinas (Fig. 2A) compared to uveitic retinas (Fig. 2B) showed destruction of the multilayered retina, especially a loss of photoreceptor outer segments in uveitic eyes (Fig. 2B). In healthy equine retinas, fibronectin immunoreactivity was detected solely in a smooth thin band along the inner limiting membrane (ILM) (Fig. 2C). Staining against fibronectin in uveitic retinas showed a disintegration of the ILM that resulted in fibronectin expression in a broad band extending towards the vitreous (Fig. 2D). Additionally, fibronectin expression was observed at the level of the outer limiting membrane as well as in isolated dotted structures throughout the tissue (Fig. 2D).

### Osteopontin is expressed along retinal Müller cells and downregulated in uveitic retinas

Osteopontin was shown to be expressed in retinal ganglion cells within the mouse retina and this expression pattern, as well as the intensity, persisted in mice with interphotoreceptor retinoid-binding protein (IRBP) induced experimental autoimmune uveitis (EAU) [27]. However, in a recently published study we demonstrated that the secreted glycoprotein osteopontin was expressed in retinal Müller cells of mouse retinal explants [22]. Therefore we were interested in further characterizing osteopontin expression within the normal equine retina and investigate the potential changes during autoimmune uveitis. Immunolabelling of osteopontin in healthy retinas revealed osteopontin expression in a Müller cell characteristic pattern including staining at the ILM. Additionally, osteopontin expression was observed in the area where photoreceptor outer segments and microvilli of the retinal pigment epithel (RPE) intertwine (Fig. 2E). Within uveitic equine retinas however, the expression of osteopontin almost disappeared (Fig. 2F) compared to control sections (Fig. 2E). A distinct positive immunoreactivity was only detected at the outer limiting membrane where photoreceptor outer segments mostly perished (Fig. 2F).

### Fibronectin closely neighbours Müller cell endfeet at the ILM and osteopontin co-localizes with vimentin in the equine retina

In order to further characterize the expression patterns of fibronectin and osteopontin within the equine retina, we performed triple labellings of healthy and uveitis diseased retina sections with the Müller cell marker vimentin. In healthy retinas, vimentin was characteristically expressed throughout the entire Müller cell from endfeet that cover the ILM to branches that pass through the inner (INL) and outer nuclear layer (ONL) to terminate at the outer limiting membrane (OLM) (Fig. 2G). In uveitic retina sections (Fig. 2H), a characteristic vimentin upregulation indicates Müller cell gliosis as it occurs in autoimmune uveitis [3], [20]. This is associated with loss of the characteristic morphology and a partial detachment of the Müller cell endfeet from the ILM (Fig. 2H). Triple labelling of healthy retina sections (Fig. 2I) revealed a closely neighbouring expression of fibronectin and vimentin at the ILM that abuts Müller cell endfeet. Futhermore, a co-localisation of osteopontin and vimentin was seen especially at the Müller cell endfeet. In uveitic retinas (Fig. 2J) the gliotic Müller cells apparently lost osteopontin expression. The spotted fibronectin expression at the disintegrated ILM does not show close association to the partly disbanded Müller cell endfeet membranes anymore (Fig. 2J).

### Primary equine Müller cells and eqMC-7 cells express osteopontin and fibronectin

**Figure 3:**
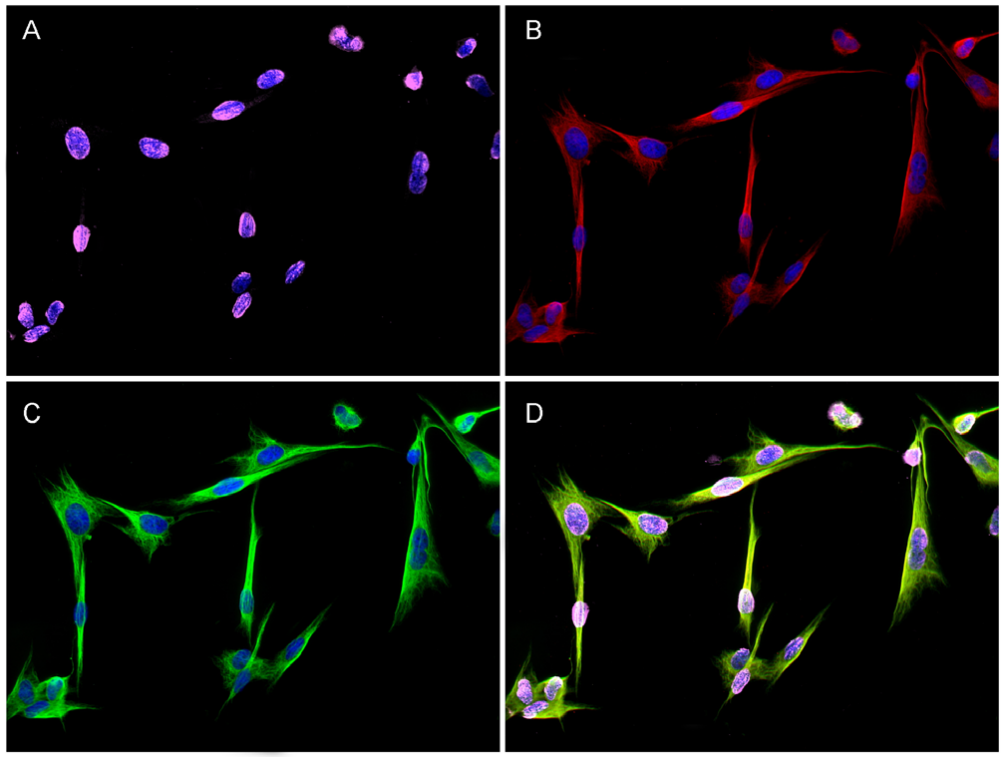
Immunocytochemical staining of cultured equine Müller cells. Representative image for eqMC-7 cells and primary cultured equine Müller cells stained for fibronectin (magenta), osteopontin (red) and vimentin (green). Fibronectin shows positive immunoreactivity in a spotted pattern predominantly in the nucleus and the surrounding area (A). Osteopontin is expressed in a thread-like distribution (B). Immunoreactivity for the intermediate filament vimentin displays an elongated, thread-like expression pattern throughout the entire cell (C). Overlay image reveals partly co-localisation of fibronectin and osteopontin in the nucleus surrounding area and co-localisation between osteopontin and vimentin stainings within the equine Müller cell (D). Nuclei are stained with DAPI (blue).

In order to verify the Müller cell associated osteopontin immunoreactivity observed in the retina (Fig. 2), and to evaluate the fibronectin expression at the ILM (Fig. 2), we performed immunocytochemical stainings of primary cultured equine Müller cells and the previously established equine Müller cell line-7 (eqMC-7) [26]. Cells were grown on glass slides and immunolabelled for fibronectin, osteopontin and the Müller cell marker vimentin. Cultured equine Müller cells showed a postitive immunoreactivity with the fibronectin-specific antibody (Fig. 3A). The expression was displayed in a spotted pattern that appeared predominantly at the nucleus and the surrounding area (Fig. 3A). Cultured equine Müller cells stained positive for osteopontin in a distribution similar to that of vimentin (Fig. 3B). Cultured Müller cells showed strong immunoreactivity for the intermediate filament vimentin in an elongated, thread-like expression pattern that appeared throughout the entire cell and is characteristic for Müller cells (Fig. 3C) [26]. Overlay image (Fig. 3D) revealed the co-localization of fibronectin and osteopontin within the equine Müller cell. Similar expression patterns of vimentin, osteopontin and fibronectin were found for eqMC-7 cells (data not shown).

## Discussion

A comprehensive understanding of autoimmune uveitis pathogenesis remains as yet elusive [6]. Analysis of differentially expressed proteins in target tissues of healthy and diseased condition provides a basis for analysis of pathogenesis-associated processes [28], [29]. To this end, proteomic studies and subsequent evaluation have revealed several enlisted proteins, biomarkers and pathways in autoimmune uveitis contributing to a better understanding of disease development and progression [28], [29], [30], [31]. In a specific comparative proteomic analysis of retinal membrane protein enriched fractions, fibronectin, a protein of the ECM, was found to be upregulated in ERU [3]. In order to evaluate this finding and further characterize its role in uveitis pathogenesis, we investigated the expression of fibronectin in vitreous and retina of control and uveitic samples since the vitreous body and the retina have been shown to play a crucial role in the pathogenesis of autoimmune uveitis [3], [28], [29]. In the present study we demonstrated a significant downregulation of fibronectin in vitreous samples of ERU diseased horses compared to controls by western blotting experiments (Fig. 1A). Furthermore, we showed a positive immunoreactivity for fibronectin exclusively at the ILM of healthy equine retinas (Fig. 2C). This expression varied in uveitic retinas displaying a disintegrated pattern along the ILM expanding towards the vitreous (Fig. 2D). With these results we could again confirm that comparative proteomic analysis is a reliable tool to reveal expression changes of molecules and can provide a basis to suggest pathways potentially involved in disease pathogenesis when validated and further investigated. Fibronectin is a ligand to various intergrin receptors and thus mediates adhesion and interaction of cells with the ECM [32]. The expression of fibronectin within the retina was investigated in several studies and inconsistent results were presented. While some immunohistochemical studies on retinal tissue of human eyes failed to detect fibronectin [33], [34], studies in rat and human retinas demonstrated positive fibronectin immunoreactivity at the ILM [35], [36]. The ILM is the vitreo-retinal border that represents the connection between the retina and the vitreous body and was shown to be built by retinal Müller cells that abut this basement membrane with their endfeet [37], [38]. In the present study immunohistochemical stainings of cultured equine Müller cells and eqMC-7 cells confirmed the ability of Müller cells to express fibronectin (Fig. 3A). It was reported that fibronectin is implicated in adhesion of cultured rat Müller cells [19]. Therefore, our results indicate that fibronectin might be produced by Müller cells to anchor their endfeet membranes to the ILM and thus contribute to the attachment of the retina to the vitreous body. Müller cells exhibit a special morphology spanning the entire retinal thickness from the ILM to the OLM that is essential for many of their functions [39]. Recently, a novel function of Müller cells was described that is facilitated by their special funnel shape morphology. Müller cell endfeet that cover the entire inner retinal surface, enable a highly efficient entry of light into the cell to conduct light through the inverted retina, preventing light scattering. Thus, depending on their special morphology, Müller cells act as optical fibres [40]. Autoimmune uveitis is associated with an extensive disintegration of the ILM (Fig. 2B) and a loss of the characteristic Müller cell morphology including detachment from the ILM (Fig. 2H). This detachment could result from the changed expression pattern of fibronectin in uveitic retinas compared to healthy controls (Fig. 2C and 2D), since fibronectin was shown to mediate Müller cell adhesion. The even expression of fibronectin along the ILM and therefore the connection to Müller cell endfeet is demolished in uveitis. This might also affect the entry of light into the cell, representing a link to a reduced or even a loss of light conductance and thus vision, which has been reported to be a consequence of uveitis [41].

Another very important function of Müller cells within the retina is neuroprotection that is performed by the release of neurotrophic factors as well as by providing various functions that support neurons in nutrition and metabolism [42]. Recently, we demonstrated that osteopontin expression in mouse retinal sections is co-localized to Müller cells and osteopontin is also secreted from cultured mouse Müller cells. Furthermore, a positive survival effect of osteopontin on cultured porcine photoreceptors and reduced apoptosis in organotypic cultures of retinas derived from rd1 mice, which carry a mutation in the photoreceptor-specific PDE6b gene, demonstrated that osteopontin acts neuroprotective [22]. In order to further evaluate the role of osteopontin in another retinal disorder, we chose to investigate osteopontin in vitreous and retina of autoimmune uveitis affected equine eyes. A study in eyes from humans affected with primary open-angle glaucoma reported a reduced osteopontin level in vitreous when compared to controls [43]. However, vitreous of patients with diabetic retinopathy showed increased osteopontin levels [44]. In the present study, we showed a significant downregulation of osteopontin in uveitic vitreous compared to controls (Fig. 1B and 1C). A double band at the molecular weight of 30 kDa (Fig. 1B) and a band triplet at the molecular weight of 72 kDa (Fig. 1C) were detected. This is in agreement with other studies and represents highly variable posttranslational modifications of osteopontin [43], [45], [46]. In healthy equine retinas, osteopontin showed a characteristic Müller cell expression (Fig. 2E and 2I) that was verified in cultured equine Müller cells (Fig. 3B). This finding confirms our previous result in mouse retinas [22]. In contrast, several studies in mouse, rat and human retinas demonstrated an immunoreactivity of osteopontin in retinal ganglion cells, but no expression in Müller cells was described [27], [47], [48]. Here, we additionally found a positive osteopontin reaction in the area where photoreceptor outer segments and microvilli of the RPE intertwine (Fig. 2E). No reports about the expression of osteopontin in photoreceptors are available at present whereas expression of osteopontin in the retinal pigment epithelial cell line ARPE-19 was shown by flow cytometry [49]. Additionally, the expression of osteopontin mRNA was detected in cultured bovine RPE cells [50]. Therefore, the positive immunoreactivity found here, could display microvilli of the RPE merging into photoreceptor outer segments.

In the present study, a loss of osteopontin expression in Müller cells of retinas from horses with autoimmune uveitis was evident (Fig. 2F and 2J). Müller cells undergo gliotic alteration in autoimmune uveitis that is associated with changed expression levels of a set of proteins [20], [28]. Gliosis represents a cellular attempt to protect the tissue from further damage. However, neuroprotective effects of gliosis oppose its detrimental consequences [51]. Therefore, the expression of osteopontin in Müller cells might represent a neuroprotective attempt that gets lost in autoimmune uveitis and thus is associated with severe neuronal damage. Besides our recent report demonstrating the neuroprotective effect of osteopontin [22], several other studies revealed a neuroprotective potential of osteopontin. Reduced cell death was shown in cortical neuron cultures deprived of glucose and oxygen and incubated with osteopontin and intracerebral administration of osteopontin caused a reduction of infarct size in a murine stroke model [21]. Osteopontin-deficient mice exhibited increased thalamic neurodegeneration following induction of cortical stroke [52]. Furthermore, apoptosis in a rat model of hypoxia-ischemia neonatal brain injury was reduced by osteopontin application [53]. However, studies in IRBP induced EAU in wild-type and osteopontin-deficient mice demonstrated increased osteopontin immunoreactivity in wild-type mice and attenuated disease in osteopontin-deficient mice, assuming a proinflammatory effect of osteopontin [23], [27]. Blockade of osteopontin with small interfering RNA confirmed the reduced clinical and histopathological scores in an EAU mouse model compared to controls [24]. The significant downregulation of osteopontin in vitreous and retinal Müller cells in the spontaneous animal model of autoimmune uveitis studied here, however, indicates a reduced neuroprotection potential of Müller cells, thus reinforcing the neuroprotective potential of osteopontin.

In uveitic equine retinas an expression of both, osteopontin and fibronectin was additionally detected in a rough line aside from the mostly disintegrated outer limiting membrane (Fig. 2D, 2F, 2J). Since photoreceptor outer segments degenerate in autoimmune uveitis, this finding raises the question whether the osteopontin and fibronectin positive structures belong to retinal tissue or to the adjacent retinal pigment epithelium. Two studies demonstrated the ability of cultured RPE cells to express osteopontin [49], [50], however, reports about RPE derived osteopontin in retinal disorders are currently not available. Although we didn't detect fibronectin in healthy RPE, other studies showed that fibronectin is expressed by RPE cells and promotes the adhesion of RPE to the ECM [54], [55]. Furthermore, an increased expression and secretion of fibronectin by RPE cells was demonstrated in presence of human serum [56]. Vascular leakage is a characteristic incident that occurs with autoimmune uveitis-related breakdown of the blood-retinal barrier [28]. Therefore, additional investigations on the expression of osteopontin and fibronectin within the retinal pigment epithelium in association with autoimmune uveitis are needed to further assess their role in disease pathogenesis.

Investigations of molecular mechanisms in autoimmune uveitis are mainly focused on cell-associated proteins, whereas the knowledge about ECM protein expression in disease pathogenesis is limited. We aimed to determine the role of ECM in autoimmune uveitis-related tissues by investigating expression profiles of relevant ECM proteins. The results indicate severe ECM re-modeling in autoimmune uveitis highlighted by reduced neuroprotection mediated by Müller cell-derived osteopontin and a loss of Müller cell adhesion through fibronectin. Thus the investigation of ECM proteins is particularly worthwhile to gain further insight into the molecular pathomechanisms occurring in autoimmune uveitis affected tissues. The impact of ECM re-modeling and its consequences for the surrounding tissue in autoimmune uveitis merits further investigation.
